# The impact of various daily disposable contact lens materials on contrast sensitivity

**DOI:** 10.5935/0004-2749.2025-0015

**Published:** 2025-06-24

**Authors:** Burcu Nurözler Tabakci, Seda Duran Güler, Gül Varan, Petek Aksöz, Yusuf Yildirim

**Affiliations:** 1 Department of Ophthalmology, University of Health Sciences, Basaksehir Cam and Sakura City Hospital, Istanbul, Turkey; 2 Department of Ophthalmology, Istanbul Medipol University, Faculty of Medicine, Istanbul, Turkey

**Keywords:** Contact lenses, Contrast sensitivity, Astigmatism, Lighting, Visual acuity

## Abstract

**Purpose:**

This study aimed to compare the effects of three different daily disposable contact lens materials on contrast sensitivity.

**Methods:**

The participants were aged 18-45 years, with spherical equivalent refraction between -0.50 D and -6.00 D, astigmatism below 0.75 D, and best contact lens-corrected visual acuity of 0.0 logMAR or better. Each patient was fitted binocularly with three daily disposable contact lenses made of different materials on three separate examination days. These materials were kalifilcon A, senofilcon A, and verofilcon A. The contrast sensitivity of each patient was recorded at spatial frequencies of 3, 6, 12, and 18 cycles per degree (cpd) under photopic (85 cd/m^2^) and mesopic (3 cd/m^2^) conditions.

**Results:**

The current study comprised 72 eyes of 34 female and two male patients. The mean age of the participants was 25.63 (± 0.80) years. Under photopic conditions, the participants’ contrast sensitivity was significantly better with senofilcon A than with kalifilcon A at a frequency of 12 cpd (p=0.008). Under mesopic conditions, participants’ contrast sensitivity was significantly higher with kalifilcon A than verofilcon A at 3 cpd (p=0.001), and with senofilcon A than verofilcon A at 12 cpd (p=0.004). The pre-lens non-invasive break-up times did not differ significantly between the three daily disposable contact lenses (p>0.05).

**Conclusion:**

In both photopic and mesopic lighting conditions, the participants in this study exhibited differences in contrast sensitivity when wearing three different daily disposable contact lens types, despite similar visual acuity and pre-lens tear film stability results in their clinical evaluations. These findings demonstrate the potential for subjective visual complaints arising from variations in the contrast sensitivity achieved by different daily disposable contact lenses.

## INTRODUCTION

Disposable silicone hydrogel contact lenses (CLs) for daily use are the trusted choice of eye care professionals for correcting refractive errors while ensuring safety and comfort^(1^,^2)^. The clarity of vision and dryness are important factors determining patient satisfaction in the long-term continuity of contact lens wear^(3)^. Other factors include the contact lens replacement period, the design of the lens edge, and lens movement^(4)^. Various materials with different surface technologies have been used to develop CLs that offer satisfactory vision correction and comfort throughout the day. Currently, the high oxygen permeability of silicone hydrogel material has led to more satisfactory results when combined with daily use^(5)^.

Despite 20/20 visual acuity in routine eye examinations, many CL wearers have subjective visual complaints^(6)^. Visual acuity is just one aspect of visual function, and its assessment does not provide a full picture of the quality of vision. The Snellen chart commonly used in clinical evaluations is a standardized approach to measuring visual acuity. This evaluates visual acuity at 100% contrast using black letters on a white background. However, the visual elements of our everyday environment include objects of various sizes with differing degrees of contrast^(7)^.

The contrast sensitivity test is a validated method for assessing visual function that reflects our real-world visual experiences. Contrast threshold is the ability to discriminate the lowest difference in illumination between the object and the background. The size of an object affects the amount of contrast needed to distinguish it from the background. The density of contiguous dark and light lines within a bounded visual field is termed spatial frequency^(8)^. Each item on a contrast sensitivity test presents a different spatial frequency and contrast level. The Snellen chart evaluates different spatial frequencies since each letter has a different shape. The letter sizes in the 2/10 row correspond to contrast sensitivity in the range of 4-6 cpd, while the letter sizes in the 20/20 row correspond to 18-24 cpd^(8^,^9)^. Patients can identify small and high contrast letters on the Snellen chart, even if there is a loss of contrast sensitivity due to any cause.

CLs divide the tear film layer of the eye, alter the distribution of the lipid layer, and impair tear film stability^(10)^. Pre-lens tear film (PLTF) stability and the wettability of the front surface of a CL are both important to visual quality^(11)^. In addition to causing unfavorable visual outcomes, impairment of the tear film causes dryness, which reduces CL conformity to the eye^(12)^. The digital screens widely used today on smartphones and computers can also cause discomfort in lens users by inducing evaporative dry eye due to inadequate blink rates^(13)^. In patients who suffer from dry eyes while wearing CLs, PLTF stability can be evaluated using non-invasive break-up time (NIBUT) tests.

This study aims to evaluate the effects of three currently available daily disposable contact lenses (DDCLs) on contrast sensitivity and PLTF stability. Each subject completed pre-lens NIBUT and contrast sensitivity tests under photopic and mesopic conditions at the end of the 1st hour with different contact lenses in 3 separate examination days.

## METHODS

This retrospective study was conducted in accordance with the tenets of the 1964 Declaration of Helsinki and its later revisions and was approved by the Ethics Committee of Basaksehir Cam and Sakura City Hospital (KAEK/27.12.2023.691). Patients recruited for inclusion in the study were aged 18-45 years, required refractive correction and had requested DDCLs, had no other ocular pathology, had spherical equivalent refraction between -0.50 and -6.00 diopters (D), had astigmatism below 0.75 D, and had best contact lens-corrected visual acuity (BCLCVA) of 0.0 logMAR or less. Patients with dry eye, corneal disorders, cataracts, pseudophakia, infectious or inflammatory ocular disease, glaucoma, retinal disorders, amblyopia, a history of ocular surgery or trauma, and patients who were pregnant or breastfeeding were excluded. Refractive and keratometric measurements were taken using an RK-F2 Full Auto Ref-Keratometer (Canon Medical Systems Corp., Ōtawara, Tochigi, Japan). Slit-lamp biomicroscopy was performed to assess each patient’s suitability for and ensure proper fitting of the CLs. Patients eligible to wear CLs were fitted binocularly with three different DDCLs, each type on a separate examination day. The lenses used were Ultra One Day (Bausch & Lomb Inc., Rochester, NY), composed of kalifilcon A silicone hydrogel; Acuvue Oasys 1-Day with Hydraluxe (Johnson & Johnson Vision Care Inc., Jacksonville, FL, USA), composed of senofilcon A silicone hydrogel; and Precision1 (Alcon Laboratories Inc., Ft. Worth, TX, USA), composed of verofilcon A silicone hydrogel. The properties of the silicone hydrogels used in the DDCLs are presented in table 1. BCLCVA was evaluated using the Snellen chart from 6 meters. Patient scores were recorded in decimals and converted to the logMAR scale. Age, sex, biomi-croscopic examination findings, NIBUT, and pupillographic measurements (obtained using a Sirius+ topographer by CSO Inc., Florence, Italy) were recorded at baseline. The NIBUT was repeated an hour after the lenses were fitted. Refractive error was corrected with the CLs, and functional acuity contrast testing (FACT) was performed an hour later and recorded monocularly at 3, 6, 12, and 18 cpd spatial frequencies. The FACT was conducted using an Optec 6500 Functional Vision Analyzer (Stereo Optical Co. Inc., Chicago, IL, USA) under photopic (85 candela per square meter cd/m^2^) and mesopic (3 cd/m^2^) luminance conditions.

**Table 1:** Characteristics of the three daily disposable contact lenses tested in this study

Parameters	Kalifilcon A	Senofilcon A	Verofilcon A
Manufacturer	Bausch & Lomb Inc.	Johnson & Johnson Vision Care Inc.	Alcon Laboratories Inc.
Material	Silicone hydrogel	Silicone hydrogel	Silicone hydrogel
Diameter (mm)	14.20	14.30	14.20
Base curve (mm)	8.60	8.50, 9.00	8.30
Dk/t (cm^2^/s)	134x10^-9^	121x 10^-9^	100x 10^-9^
Central thickness (mm)	0.08	0.085	0.09
Core water content	55	38	51
Core modulus (MPa)	0.5	0.72	0.6
Wetting technology	Advanced MoistureSeal^®^ Technology ComfortFeel Technology	HydraLuxe^TM^by internally incorporated wetting agent PVP.	SMARTSURFACE^®^ Surface water content, >80% Surface water content gradient, 3µ
UV blocker	Class II UV blocker	Class I UV blocker	Class I UV blocker

cm= centimeters; DK/t= oxygen transmissibility; mm, millimeters; MPa= megapascal; PVP= polyvinylpyrrolidone; s= second; UV= ultraviolet.

Statistical analysis was performed using SPSS for Windows, v. 26.0 (SPSS Inc., Chicago, IL, USA). The normal distributions of quantitative data were assessed using the one-sample Kolmogorov-Smirnov test. The Friedman test was used to identify significant differences between non-normally distributed variables. P-values <0.05 were considered statistically significant. When the Friedman test found statistically significant differences between the three CL types, further pairwise comparisons were made using Bonferroni-corrected Wilcoxon tests. For these, p<0.017 was regarded as statistically significant.

## RESULTS

There were 36 participants in this study, 34 females and two males, amounting to 72 eyes. The mean age was 25.63 (± 0.80, range 18-44) years. The mean refractive error was -2,45 (± 1.12, -0.5- -5.25) D, and the mean BCLCVA was 0.0 logMAR. The mean NIBUT before CL fitting was 10.53 (± 4.98, 5-17). An hour after CL application, the mean NIBUT was 5.60 (± 4.48,1.20-17) with kalifilcon A, 5.33 (± 4.67,1.20-17) with senofilcon A, and 5.82 (± 5.05, 1.20-17) with verofilcon A. For all three DDCLs, the NIBUT values were significantly lower after an hour than before lens fitting (all p<0.001). There were no significant differences between the pre-lens NIBUT values of the three different DDCLs (p=0.77). The mean pupil diameters were 5.24 (±0.94), 5.83 (±0.95), and 5.92 (±1.01) under the photopic, mesopic, and scotopic conditions, respectively.

There were no statistically significant differences between the three different DDCLs at spatial frequencies of 1.5, 3, 6, and 18 cpd under photopic conditions (p=0.391, p=0.248, p=0.130, p=0.141, respectively). Contrast sensitivity was significantly higher with seno-filcon A compared to kalifilcon A at a frequency of 12 cpd under photopic conditions (p=0.008). There was no significant difference between kalifilcon A and verofilcon A (p=0.446) or between senofilcon A and verofilcon A (p=0.021) (Table 2). There were no significant differences between the three different DDCLs at spatial frequencies of 1.5, 6, or 18 cpd under mesopic conditions (p=0.102, p=0.065, p=0.078). At 3 cpd under mesopic conditions, there was a statistically significant difference in contrast sensitivity with verofilcon A compared with kalifilcon A (p=0.001). There was no statistically significant difference between senofilcon A and kalifilcon A and between senofilcon A and verofilcon A at 3 cpd under mesopic conditions (p=0.632, p=0.028). At a frequency of 12 cpd, contrast sensitivity with senofilcon A was statistically significantly higher compared to verofilcon A (p=0.004), while there was no significant difference between senofilcon A and kalifilcon A (p=0.024) nor between verofilcon A and kalifilcon A (p=0.409) (Table 3).

**Table 2:** The contrast sensitivity of participants wearing contact lenses composed of different silicone hydrogels under photopic luminance conditions

	1.5 cpd	3 cpd	6 cpd	12 cpd	18 cpd
Kalifilcon A (mean ± SD)	1.53 ± 0.15	1.75 ± 0.15	1.78 ± 0.21	1.39 ± 0.22	0.98 ± 0.23
Senofilcon A (mean ± SD)	1.55 ± 0.15	1.78 ± 0.17	1.80 ± 0.18	1.48 ± 0.20	1.03 ± 0.25
Verofilcon A (mean ± SD)	1.52 ± 0.21	1.78 ± 0.20	1.75 ± 0.23	1.41 ± 0.22	0.95 ± 0.28
p-value	0.391	0.248	0.130	0.014^*^	0.141

* Friedman test, p<0.05 significance level.

cpd= cycles per degree; SD= standard deviation.

**Table 3:** The contrast sensitivity of participants wearing contact lenses composed of different silicone hydrogels under mesopic luminance conditions

	1.5 cpd	3 cpd	6 cpd	12 cpd	18 cpd
Kalifilcon A (mean ± SD)	1.69 ± 0.15	1.80 ± 0.14	1.63 ± 0.22	1.12 ± 0.29	0.50 ± 0.25
Senofilcon A (mean ± SD)	1.68 ± 0.18	1.79 ± 0.17	1.66 ± 0.18	1.20 ± 0.22	0.59 ± 0.26
Verofilcon A (mean ± SD)	1.64 ± 0.17	1.73 ± 0.22	1.61 ± 0.25	1.09 ± 0.30	0.53 ± 0.24
p-value	0.102	0.009^*^	0.065	0.039^*^	0.078

* Friedman test, p<0.05 significance level.

cpd= cycles per degree; SD= standard deviation.

## DISCUSSION

**Figure 1:**
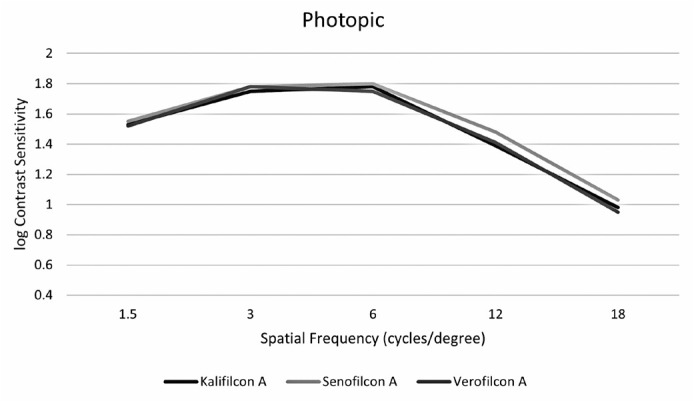
Participants’ contrast sensitivity with three types of daily disposable contact lenses under photopic conditions

**Figure 2:**
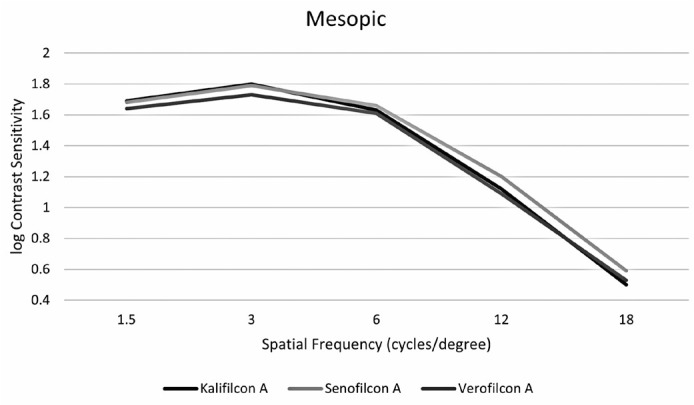
Participants’ contrast sensitivity with three types of daily dis-posable contact lenses under mesopic conditions

In this study, the novel daily disposable lens material kalifilcon A was compared with the commercially available materials senofilcon A and verofilcon A in terms of PLTF lens tear film stability and contrast sensitivity. We found no significant differences in PLTF stability between the three different CL materials. Under photopic conditions, senofilcon A demonstrated higher contrast sensitivity at high spatial frequencies. However, the difference was only statistically significant when it was compared to kalifilcon A only at 12 cpd (Figure 1). Under mesopic conditions, contrast sensitivity was lower with verofilcon A at low frequencies and higher with senofilcon A at high frequencies. It was significantly higher with kalifilcon A at 3 cpd and with senofilcon A at 12 cpd than with verofilcon A (Figure 2).

Since their invention, the comfort and visual clarity achieved with CLs have progressively improved due to the development of new materials and designs. In recent years, user preferences have shifted toward DDCLs constructed with silicone hydrogel materials^(2)^. The structure and stability of the PLTF are critical to clear vision and comfort that lasts throughout the day^(11)^. Advanced materials and surface coating technologies have been developed to minimize interaction between CLs and the tear film and maximize PLTF stability. Kalifilcon A is a novel material used in Ultra One Day (Bausch & Lomb) lenses with Advanced MoistureSeal and ComfortFeel technologies. Advanced MoistureSeal technology uses a two-step polymerization process to create a flexible silicone matrix with a combination of hydrophilic components. The moisture retention capacity is further increased by a polyvinylpyrrolidone (PVP) polymer that grows around and through the silicone matrix. Comfort-Feel technology is a combination of osmoprotectants, electrolytes, and moisturizers that are released during lens wear to help protect and stabilize the tear film^(14)^. Senofilcon A with HydraLuxe Technology (Acuvue Oasys 1-Day, Johnson & Johnson Vision Care Inc., Jacksonville, FL) is a well-known silicone hydrogel DDCL for several years. They incorporate HydraLuxe technology, which is a mesh of tear-like molecules and silicone that mimics mucin and helps to support the stability of the tear film^(15)^. The SMARTSURFACE technology used with the verofilcon A in Precision1 (Alcon Laboratories Inc.) lenses is a 2-3 micron thick permanently moist layer on the lens surface. This is >80% water content and consists of bonded hydrophilic polymers (polyacrylic acid) that crosslink with wetting agents (polyamidoamine epichlorohydrin and polyacrylamide-polyacrylic acid)^(16)^. Penbe et al. compared the effect of DDCLs (verofilcon A, senofilcon A, and nesofilcon A) on PLTF stability in healthcare workers who used face masks throughout the day during the COVID-19 pandemic. They reported that the 1st hour NIBUT values were higher than the baseline values and there was no difference between the DDCLs at the 1st hour. In this study group, no statistically significant difference was detected between senofilcon A and verofilcon A in terms of NIBUT results from baseline to the 8th hour with the use of face mask which negatively affects tear film stability. However, after 12 hours, the NIBUT values were superior with verofilcon A to those with senofilcon A^(17)^. In the present study, we found no significant differences in NIBUT values after an hour of lens wear between the three DDCLs. In our cohort, the baseline NIBUT values were significantly higher than the 1-hour NIBUT values.

Although the visual performance of CLs is critical for wearers, there has been a dearth of studies evalua-ting their effects on contrast sensitivity. Sapkota et al. investigated the effects of the material properties and wearing time of soft CLs on contrast sensitivity. DDCLs (stenofilcon A, nelfilcon A, nesofilcon A) were applied to one eye and monthly change CLs (lotrafilcon B, comfilcon A, balafilcon A) were applied to the other eye and compared. The study found no significant differences in the contrast sensitivity scores at baseline and after 3 months of CL wear. They also found no significant differences between CLs that were replaced daily and those replaced monthly or between those made with silicone and those made with hydrogel materials^(7)^.

Another study evaluated contrast sensitivity in DDCL wearers under mesopic conditions and found no difference between senofilcon A and verofilcon A at low spatial frequencies but at 18cpd spatial frequency, contrast sensitivity of verofilcon A was higher than senofilcon A (p=0.037)^(17)^. Although the difference was reported to be statistically significant, statistical significance may be possible if the *p* value was below 0.017 in the evaluation of 3 different DDCLs with post-hoc evaluation with Bonferroni correction^(18)^. In our study, no significant difference was found among senofilcon A and verofilcon A at 18cpd spacial frequency under mesopic conditions. We found that contrast sensitivity with kalifilcon A was significantly higher than with verofilcon A at 3 cpd and with senofilcon A than verofilcon A at 12 cpd under mesopic conditions.

Contrast sensitivity changes during CL wear are thought to be related to the properties of the CL material and physical changes in the CL or cornea. A study comparing the contrast sensitivity changes of seven different daily disposable CLs over a 12-hour wearing time reported no significant contrast sensitivity variation across the wearing time of each lens^(19)^. The same study found that the CLs with the lowest water content, narafilcon A (46%) and filcon II 3 (56%), produced poorer CS performance^(19)^. Grey has also reported reductions in contrast sensitivity corresponding to decreases in lens water content^(20)^. In contrast, our study found the highest contrast sensitivity performance at most spatial frequencies in senofilcon A, which had the lowest water content of 38%.

In this study, contrast sensitivity measurements were performed with the Optec-6500 device. The OPTEC-6500 (Stereo Optical, Chicago, IL) is an instru-ment in which illumination, distance, and glare are both standardized and the integrated graphics’ orienta-tion needs to be defined manually. Jung et al. compared manual (Optec 6500) and automated (CGT-2000) CS devices and found that manual CS devices showed better performance than automated ones. Still, both exhibited moderate to good test reproducibility and intertest correlation^(21)^.

Although CL wear is known to induce changes in corneal physiology throughout the day, we only assessed contrast sensitivity and PLTF stability at baseline and after one hour of CL wear^(7^,^22)^. Therefore, the lack of contrast sensitivity and PLTF stability assessments after the lenses had been worn for a full day was a limitation of this study.

In the present study, although visual acuity and pre-lens tear film stability results were similar in clinical evaluation, contrast sensitivity differences were found under photopic and mesopic conditions with 3 different daily disposable silicone hydrogel lenses. Such differences in contrast sensitivity may potentially lead to subjective visual complaints.

## Data Availability

The data that support the findings of this study are not openly available and are available from the corresponding author upon reasonable request.
